# Belt Tear Detection for Coal Mining Conveyors

**DOI:** 10.3390/mi13030449

**Published:** 2022-03-17

**Authors:** Xiaoqiang Guo, Xinhua Liu, Hao Zhou, Rafal Stanislawski, Grzegorz Królczyk, Zhixiong Li

**Affiliations:** 1 School of Mechatronic Engineering, China University of Mining & Technology, Xuzhou 211006, China; godric_guo@cumt.edu.cn (X.G.); 0219@csit.edu.cn (H.Z.); 2 School of Intelligent Manufacturing, Suzhou Chien-Shiung Institute of Technology, Taicang 215400, China; 3 Department of Electrical, Control and Computer Engineering, Opole University of Technology, 45-758 Opole, Poland; r.stanislawski@po.edu.pl; 4 Faculty of Mechanical Engineering, Opole University of Technology, 45-758 Opole, Poland; g.krolczyk@po.edu.pl (G.K.); zhixiong.li@yonsei.ac.kr (Z.L.)

**Keywords:** machine vision, deep learning, smart mining, smart cities

## Abstract

The belt conveyor is the most commonly used conveying equipment in the coal mining industry. As the core part of the conveyor, the belt is vulnerable to various failures, such as scratches, cracks, wear and tear. Inspection and defect detection is essential for conveyor belts, both in academic research and industrial applications. In this paper, we discuss existing techniques used in industrial production and state-of-the-art theories for conveyor belt tear detection. First, the basic structure of conveyor belts is discussed and an overview of tear defect detection methods for conveyor belts is studied. Next, the causes of conveyor belt tear are classified, such as belt aging, scratches by sharp objects, abnormal load or a combination of the above reasons. Then, recent mainstream techniques and theories for conveyor belt tear detection are reviewed, and their characteristics, advantages and shortcomings are discussed. Furthermore, image dataset preparation and data imbalance problems are studied for belt defect detection. Moreover, the current challenges and opportunities for conveyor belt defect detection are discussed. Lastly, a case study was carried out to compare the detection performance of popular techniques using industrial image datasets. This paper provides professional guidelines and promising research directions for researchers and engineers based on the leading theories in machine vision and deep learning.

## 1. Introduction

With the development of industrial automation, conveyor belts have been used as one of the most significant pieces of equipment for the transportation of various industrial products. The belt-strengthened conveyor, with the advantages of simple structure, low cost and high carrying capacity, is widely used in the energy and mining industries. However, factors such as aging by long-term operation, heavy or impact loads, complex operating environments and long transport distance result in adverse conveyor belt phenomena, even wear and puncture. Undetected worn or punctured spots on conveyor belts can develop into long-range tears. No matter how short the unexpected downtime is, the company still suffers from huge financial losses. Furthermore, some kinds of serious emergencies caused by conveyor belt failure may lead to a major accident in mining enterprises.

In order to explore the factors of belt failure and research conveyor belt tear detection methods in depth, we studied the basic composition and structure of conveyor belts. Conveyor belts, which consist of several composite layers, even steel cord, are commonly used in the mining industry, mainly to transport heavy loads of ore and coal. The complex composition and structure of conveyor belts makes tear detection difficult, and extra preparatory work for failure inspection is required for different detection methods.

Considering long-distance transportation and the harsh underground environment, comprehensive manual inspection of conveyor belts is a tough and tedious task. With the improvement of industrial inspection and the development of high-sensitivity sensors, non-destructive testing (NDT) methods have drawn more and more attention. Considering the different sensors and algorithms, NDT methods can be divided into several categories: sensor-based methods, X-ray/multispectral-based methods and 2D/3D image-based methods. For sensor-based methods, conveyor belts are redesigned or modifications are made to provide detecting sensors with special signals. For other NDT methods, various acquisition devices, such as CCD cameras, X-ray emitters/receivers, multispectral sensors, etc., are installed to acquire the necessary data. Nowadays, NDT methods based on abundant data acquired by various sensors possess incomparable advantages and have become mainstream techniques.

In this article, we reviewed 90 papers collected from SciVerse, ScienceDirect (http://www.sciencedirect.com/), Springer Link (http://link.springer.com/) and IEEE Xplore Digital Library (http://ieeexplore.ieee.org) and the reference lists of contained articles. The reviewed papers were published since 1980s and available online before May 2021 and discuss the development of inspection methods for conveyor belt tears. Starting with the basic structure of the conveyor belt, we will discuss the advantage and shortcomings of various tearing detection methods. The contribution of this paper can be categorized as three parts:

Firstly, we study the basic structure of conveyor belts and classify the causes of common conveyor belt tears.

Secondly, we review the mainstream techniques and theories for conveyor belt tear detection and discuss their advantages, shortcomings and characteristics.

Thirdly, we survey the datasets of acquisition and preparation theory, which is the most important part for supervised machine learning algorithms.

Finally, we discuss the opportunities and challenges for conveyor belt tear detection under the rapid development of information computing and processing technology.

**Figure 1:**
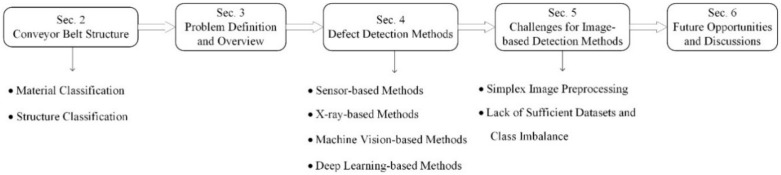
Overview of defect detection for conveyor belts.

The structure of the rest of the paper is presented in Figure 1. The common basic structures of conveyor belts are presented in Section 2. The mainstream techniques of sensor-based methods, 2D/3D image-based methods and X-ray/multispectral-based methods are reviewed in Section 3. Section 4 discusses dataset preparation for machine vision and deep learning algorithms. The future opportunities and challenges of conveyor belt surface inspection are discussed in Section 5, and Section 6 concludes the paper.

## 2. Basic Structure of Conveyor Belts

**Figure 2:**
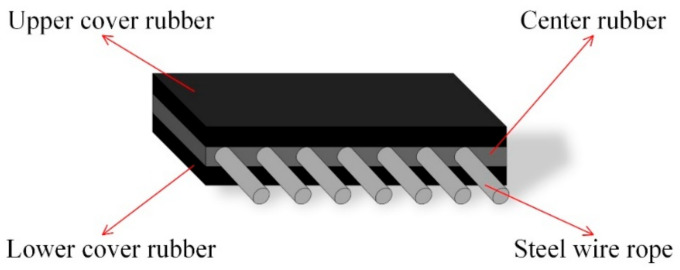
Sandwich structure of a conveyor belt crossed by steel cord.

As one of the most commonly used pieces of transportation equipment in industry, conveyor belts can be classified into several categories by type, such as food-grade conveyor belt (polyurethane, PU or polyvinyl chloride (PVC)) in the food industry; flexible-chain conveyor belts in automated industry; and reinforced multilayer conveyor belts in the mining industry. Conveyor belts with heavy loads used in power plants, the mining industry and the chemical field consist of several layers and steel cords, which are usually processed with a variety of chemical components. Chemical-treated multilayer composite belts have the advantages of high strength and wear resistance. Common conveyor belts used in underground coal transportation consist of three layers. The mid-layer crossed by steel cord is made a mixture of styrene–butadiene rubber (SBR) [1] and natural rubber (NR) [2] to gain extra adhesiveness with steel wire ropes. The upper and lower layers is made of butadiene rubber (BR) [3] to acquire abrasion resistance for long-term transportation. The sandwich structure of conveyor belts crossed by steel cord is shown in Figure 2. Normal conveyor belts are the same as the belt described above, except embedded steel cord and made of composite rubber or nylon, which leads to lower capacity and wear resistance.

Based on the basic structure, conveyor belts can be designed with different thickness and can be processed by different chemicals for application in various scenarios. The surface of conveyor belts can be molded rugged lines to increase friction. For some specialized fields, conveyor belts are embedded with coils, which results in high strength and cost. Because of the special sandwich structure of conveyor belts, scholars have come up with many methods to inspect belt tears in real time.

## 3. Defect Detection for Conveyor Belts

### 3.1. Problem Definition

The goal of conveyor belt detection methods is to identify defects on the conveyor belt surface or damages to the inside and classify these defects into several categories, such as wear damage or puncture damage. Most such failures can be distinguished by load strength and duration. The characteristics of low load strength and long duration is reflected in wear damage, which is mainly caused by cracks or scratch extension in the surface layer and does not result to unexpected failure. If wear damage accumulates or the conveyor belt suffers from a huge load impact imposed by sharp metal objects, puncture damage occurs. Differently from wear damage, the punctured spot simultaneously affects all layers of the conveyor belt and greatly weakens the belt carrying performance. If the punctured spot on the conveyor belt cannot be detected in time, it could lead to catastrophic failure. Conveyor belt deviation may cause an imbalance of the left and right sides along the length, as well as abnormal tension. Imbalanced belts are worn with frames and bearings, which could lead to belt tear in some extreme situations. Belt aging due to pool quality, lack of maintenance, overloading or overheating is another of the most significant causes of belt damage or failure. Furthermore, tear detection methods focus on the inspection of torn parts on the conveyor belt and provide some kind of immediate alarm. 

### 3.2. Mainstream Techniques

The basic structure of conveyor belts is discussed in Section 2. Various modifications may be applied to adapt to different fields. For the mining industry, the composite layers of conveyor belts are processed with special chemical treatment and most commonly embedded with steel cords. Considerable research has been conducted on conveyor belt tear detection, and the mainstream NDT detection methods can be classified into three categories: sensor-based detection methods, X-ray/multispectral-based detection methods and 2D/3D image-based and hybrid detection methods (Table 1).

### 3.3. Detecting Sensors

The leak of transported goods from the bottom of the belts often indicates the puncture damages to the conveyor belts. Hence, some sensor-based detection methods, such as LTCD, detect protrusion or leakage from the lower surface of an idle belt [4]. In general, the main idea of similar detection methods is to interpret other invalid status signals of the conveyor belt, such as belt relative width or transported material spillage or leakage, as the valid belt longitudinal tearing discriminant. These types of sensors and devices are sensitive to installed location, belt materials and parameter settings, which lead to poor adaptability.

Magnetic-based methods require some extra preparation, such as the implementation of coils and magnetized steel cords attached on different parts of the conveyor. Earlier research [5] was conducted on conveyor belt detection based on the measurement of magnetic field changes generated by injured steel cords embedded in the mid-layer. A special device records the magnetic field distribution in advance and dynamically compares the real-time magnetic field distribution to the recorded distribution. If the magnetic field differences are beyond a threshold value, it means that the belt has been torn. Derivative methods [6] based on magnetic field changes have been developed for many years and kept the essential theory untouched. G the limitation of integrated circuit and microcomputer performance, the analysis of magnetic field changes for a large span and duration is quite tricky. Although the data of each channel narrow with the increasing number of the magnetic sensors, users still need to analyze the fluctuation of each signal [7]. With the development of sensor technology and high-performance processors, methods [8,9] based on multidimensional magnetic signal acquisition and optimization have become possible. Damaged spots of conveyors belt have been detected with abundant sensors and 2D digital imaging [10,11]. These magnetic-based methods convert injured locations into sensor signals, which are visualized as 2D images in real time.

Other methods [12] based on electromagnetic interaction set up electromagnetic energy pathways from transmitter to receiver. The transmitter and the receiver are installed on the both sides of the conveyor belt surface. The electromagnetic signal emitted by the transmitter reaches to the receiver on the other side along the vulcanized conveyor belt. As long as the continuous signals are interrupted and the receiver cannot read a sufficient signal, the conveyor belt is considered torn, and the conveyor stops immediately.

#### 3.3.1. X-ray/Spectrum

As a typical NDT method, X-ray detection is widely used to construct and analyze internal images of samples. X-ray based NDT methods [13,14,15,16,17,18,19] used in online industrial inspection require special designs since X-ray has the characteristics of high penetration and can be harmful to human beings. In simple terms, the X-ray goes through the running conveyor belt, and the intensity of radiation received on the other side constantly fluctuates in a fixed range if the conveyor belt is in good condition. If parts of belt are internally damaged, the value of radiation is abnormal. A detection algorithm in an industrial computer records and analyzes data of radiation intensity and makes judgments about the state of the conveyor belt in real time.

**Figure 3:**
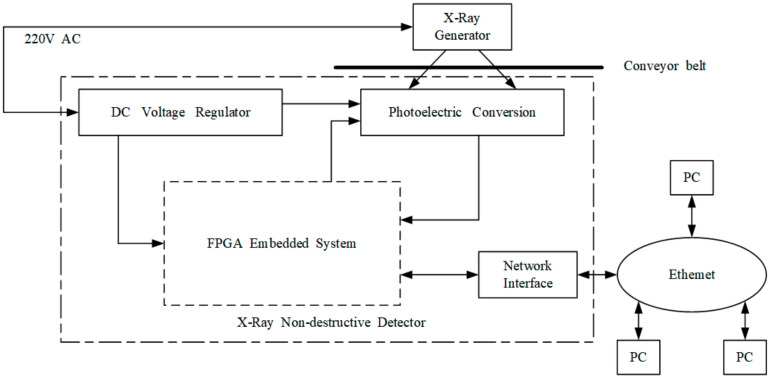
Basic hardware topology of the X-ray-based conveyor belt method.

To avoid damage to the human body, the X-ray source and X-ray detector are surrounded in lead shell. Considering the transported materials on the conveyor belt surface, the X-ray source and X-ray detector are installed on the opposite sides of the lower conveyor belt (Figure 3). Hence, in addition to the complex structural design of the X-ray radiator and receiver, the significant disadvantage is that online X-ray-based NDT methods cannot immediately detect conveyor belt tears.

To overcome the disadvantage of X-rays being harmful to human body, infrared and multispectral cameras have been studied by increasing numbers of researchers. The dusty and dark underground environment in coal mines leads to poor image quality with normal industrial cameras, which is a key problem for image feature extraction and analysis. However, the infrared light, whose wavelength is longer than those of the visible light, has strong diffraction and can penetrate the dust. It makes clear images of conveyor belt tears with CMOS sensors. Furthermore, the different layers of conveyor belts are made of different materials, the radiation wavelengths of which are distinct from each other. This primary feature makes conveyor belt wear or tear detection possible in dusty and dark environments since the mid-layer material would be exposed when the tear damage to the surface layer of the belt happens. The key points of infrared-based conveyor belt tear detection method are optical path design and image feature analysis. Yang et al. [20] made use of an infrared camera and proposed a tear detection algorithm containing image enhancement and binarization. Yang et al. [21] analyzed the belt damage process and designed a novel conveyor belt tear detection method based on infrared thermal imaging technology. With the appropriate spectral passband selected and the radiation fitting image, spectrum features are extracted from 2D spectrum signals obtained by Fast Fourier Transform. The experimental results showed significant detection performance and effectiveness. To compensate for poor image quality in underground environments, a hybrid method was proposed [22] with normal CMOS sensors and infrared sensors. A special optical path was designed to address the synchronous image-fusion issue, since the infrared image and the normal image are acquired by different devices. In the last step, the fused 2D image is processed with machine vision algorithms. Inspired by the above theory, Yu et al. [23] proposed a dual-band infrared detection method with long-infrared CCD sensors and mid-infrared CCD sensors. With several splitting films and plane mirrors, the infrared radiation from substances is split into two parts and obtained by MIR CCD and LIR CCD, respectively. To improve the image feature quality, a multispectral-based method [24] was proposed, combining far-infrared images, visible light images and mid-infrared images. A comparison of X-ray/spectrum methods is presented in Table 2.

The wavelength of visible light spans from approximately 400 nm to 780 nm, in which the images captured by CMOS cameras are poor-quality and feature-limited. Spectrum-based methods broaden the conveyer belt tear features by applying extra infrared devices. With extra infrared sensors and a special optical path, the fusion images are significantly enhanced. However, the disadvantages are also obvious: infrared devices are quite expensive, and optical path design and device installation are complex, taking up more space.

#### 3.3.2. 2D/3D Images

**Figure 4:**
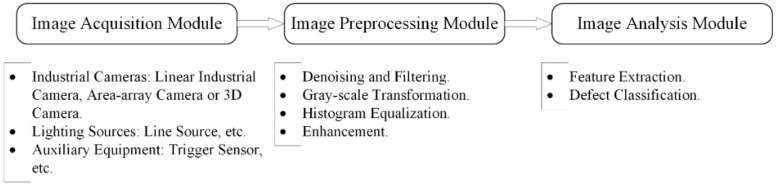
Fundamental components for image inspection system.

With the application of state-of-the-art image processing techniques on digital conveyor belt images, conveyor belt inspection has been revolutionized. Given the rapid development of artificial intelligence and computer processing speed, image-based failure detection methods have higher inspection speed and precision than other methods. Furthermore, less modification and easier maintenance of original devices make image-based methods more competitive. The basic inspection system consists of an image acquisition module, image preprocessing module and image analysis module, which are shown in Figure 4. The image acquisition module contains industrial cameras, lighting sources and auxiliary equipment, such as an encoder or trigger switch. Image preprocessing and analysis modules are handled by high-performance computers or embedded devices. The significant differences between image-based detection methods and other methods are the means of data acquisition and data processing. In contrast to other sensor-based detection methods, image-based detection methods have the characteristics of visualization and more information data for conveyor belts, which are achieved by employing high-speed CCD or CMOS cameras and high-performance computers. 

The development of image-based conveyor belt detection methods has mainly experienced two stages: machine vision-based image processing algorithms and deep learning-based image processing algorithms. The typical machine vision-based image processing algorithms consist of some basic steps, such as preparation, computation and analysis, which are carefully designed for particular targets. Hence, a lot of experience is need for machine vision engineers to complete algorithm design at each step, from equipment selection to data processing and analysis. However, as opposed to a special design for algorithms, the framework of deep learning-based image processing is designed, and the neural network is trained automatically with sample datasets prepared in advance. Compared with machine vision-based image processing algorithms, deep learning-based image processing is focused on hierarchical structure design for the higher levels and computed on more efficient GPUs with higher speed.

#### 3.3.3. Machine Vision

**Figure 5:**
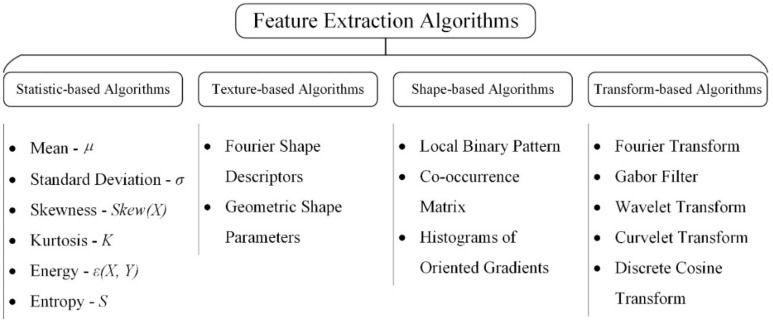
Taxonomy of feature extraction methods.

Machine vision-based methods depend on artificially designed feature extraction algorithms, which is the core part of the whole defect detection algorithm. As shown in Figure 5, feature extraction algorithms can be classified into four parts: (1) grayscale-based; (2) texture-based; (3) shape-based; and (4) transform-based algorithms.

The most useful terms for grayscale-based algorithms, which are used to reflect the statistical features, can be described by the following formulas.

(1) $$\mu =\frac{1}{l}\overset{l-1}{\underset{i=0}{\sum }}V_{i}$$

(2) $$\sigma =\sqrt{\frac{\sum_{i=1}^{n}{\left(x_{i}-\bar{x}\right)}^{2}}{n-1}}$$

(3) $$Skew\left(X\right)=E\left[{\left(\frac{X-\mu }{\sigma }\right)}^{3}\right]$$

(4) $$K=\frac{1}{n}\overset{n}{\underset{i=1}{\sum }}{\left(\frac{x_{i}-\mu }{\sigma }\right)}^{4}$$

(5) $$\varepsilon \left(X,Y\right)=2E{\left\lceil X-Y\right\rceil }_{d}-E{\left|X-X^{\prime }\right|}_{d}-E{\left|Y-Y^{\prime }\right|}_{d}$$

(6) $$S=-K\overset{n}{\underset{i=1}{\sum }}\left(p_{i}\log_{2}p_{i}\right)$$

**Figure 6:**
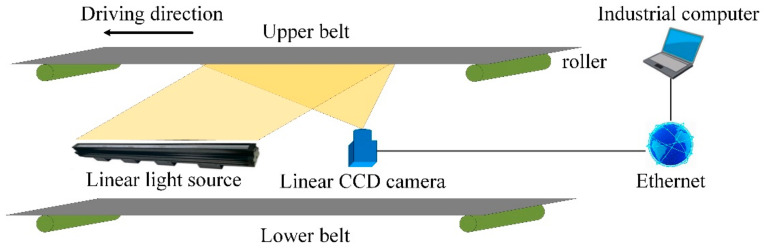
Sketch of underground image acquisition devices.

The characteristics, i.e., long span, dim and dusty underground environment and arched section of the mine conveyor belt, result in a similar equipment layout, which is shown in Figure 6. An array of several industrial cameras and light sources, high-speed data transmission cables, embedded computing units and accessories are common hardware for image acquisition and processing. In order to satisfy the requirement of real-time detection, the light sources and cameras are usually deployed between upper and lower belts and capture undersurface images of the upper belt. Being mounted in this way, cameras can obtain damage images in real time as long as tear or wear damage happens. However, the major differences in detection performance are caused by image processing strategy for various machine vision-based defect detection methods, which contain one or more processes, e.g., feature extraction methods, image enhancement methods, pattern recognition, etc.

Li et al. [25] designed a conveyor belt inspection method adopting a feature extraction algorithm, including image conversion, denoising and enhancement. Based on the theory of maximum mutual information entropy, an improved image segmentation method was proposed to obtain tearing region. Several features were empirical selected to determine the status of the belt. An improved image segmentation method targeting belt tearing and deviation was proposed in [26]. Based on the obvious difference between the gray value of belt in tearing and other parts, the average column vectors acquired by the height direction projection was adopted to identify belt edges. Rip and deviation identification were processed through the self-adaptive threshold segmentation method. Li et al. [27] analyzed fuzzy images captured underground and compared single-scale retinex (SSR) and multi-scale retinex (MSR) algorithms. A combined algorithm of morphology gradient and SSR was applied to enhance image contrast in edge regions and smooth the image in non-edge regions. A series of image processing, i.e., threshold segmentation, binarization and characteristics extraction, was adopted to make belt failure detection possible. Zeng et al. [28] proposed a feature fusion method consisting of Gaussian filtering, local image enhancement and Canny edge detection. The length of the rectangular area outlined by Canny edges and the ratio of length to width of the rectangular area are considered main geometric features. Gray template-matching level, examined by normalized coefficients between template and search graph, is chosen as similarity feature. Several failure characteristics of conveyor belt images were studied in [29], and a multi-class support vector machine (SVM) classifier was proposed to identify belt damages. An adaptive threshold method based on visual saliency enhances image contrast, decreases image noise and prepares characteristics extraction. Three types of surface damages, i.e., scratch, crack and tear, are detected with SVM classifiers. Hou et al. [30] proposed an improved image segmentation and morphological operation algorithm with aided audio analysis to detect conveyor belt tearing regions. The region growing algorithm was applied to the potential defect parts, and the segmentation result, combined with processed audio information, was examined. A conveyor belt tearing classifier based on Haar-like features and the AdaBoost algorithm was designed in [31]. The characteristics of conveyor belt tearing regions, edge features, line features and special diagonal line features were selected to construct Haar-like features. The strong classifier, which was adopted to detect tearing regions with a much higher detection accuracy, was trained and connected by a cascade algorithm. Laser-assisted methods [32,33,34] convert conveyor belt tear features into several linear laser breakages, which are captured by CMOS or CCD cameras, and then indirectly detect these breakage points by machine vision algorithms. Multiple sets of laser images were acquired in [32], and the Sobel operator with a specially shaped kernel was applied to ROI containing enhanced laser stripes to detect belt tear areas. Lv et al. [33] proposed an improved gray-gravity center (IGGM) method to determine the centerline of the laser region. The IGGM method weakens the influence of image noise and sets up the connection of gray-gravity center points in different rows. Table 3 lists the pros and cons of machine vision methods.

#### 3.3.4. Deep Learning

**Figure 7:**
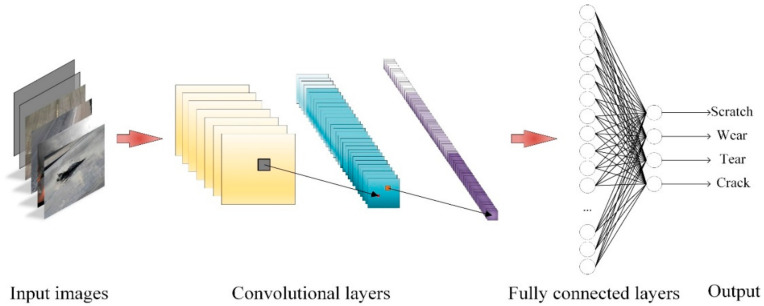
Pipeline of convolutional neural networks.

With the development of high-performance GPUs and big datasets, deep neural networks have received more and more attention from scholars, which heralds the deep learning renaissance. The essential goal of deep learning-based image processing algorithms for conveyor belts is target detection—more specifically, detection of scratches, wear or tear on conveyor belt images. Deep convolutional neural networks (DCNNs), the leading architecture of deep learning-based methods for detection or classification tasks, consist of several basic components: convolutional and pooling layers, usually grouped as modules, activation layers, fully connected layers, etc. As shown in Figure 7, the input image is classified into a certain category after weighing with convolutional, activation and fully connected layers.

Mainstream object detection methods based on deep learning are classified into two categories: two-stage methods, represented by R-CNN (region convolutional neural network) series; and single-stage methods, represented by YOLO (You Only Look Once) methods and SSD (single shot multibox detector) series. Over the last few years, many robust and accurate deep learning algorithms represented by R-CNNs have been developed.

(1) Backbone Networks

**Figure 8:**
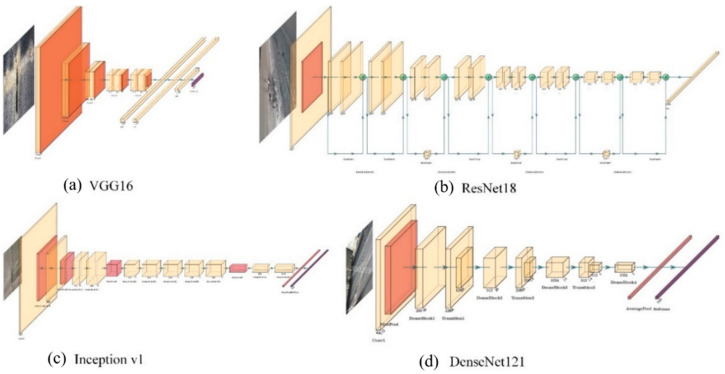
Architecture of deep neural networks: (**a**) VGG16; (**b**) ResNet18; (**c**) Inception; (**d**) DenseNet.

Backbone networks in deep learning algorithms are applied to extract features from input images. Considering computational efficiency and detection accuracy, the common backbone network is chosen from well-performing deep neural network image classifiers, except that the last layer used for classification is replaced by another specially design layer. As a result, the deep learning algorithm obtains higher detection accuracy and better computational efficiency. For certain resource constraints, network depth, width and input image resolution are the key factors, which can influence ConvNet accuracy. Besides the gradient vanish problem, it is well known that deeper neural networks have stronger feature extraction capability [35,36,37]. Another widely used technique is to expand the ConvNet width for small networks. More fine-grained features tend to be acquired in wider networks [38,39,40]. However, feature extraction capability is restrained in extremely wide but shallow networks because of higher-level feature loss. Recently, more and more ConvNets have taken high-resolution images as input, and experimental results in [41] show that image resolution increases with limited accuracy gain and reduced computational efficiency. Some favorite backbone networks with these specific characteristics are discussed as follows. Four typical backbone networks are listed in Figure 8, i.e., VGG16, ResNet18, Inception v1 and DenseNet121.

VGGNets [42] are characterized by the combination, as a basic unit, of convolutional layers with small 3 by 3 filters and a 2 by 2 max pooling layer. The difference between VGG16 and VGG19 is the depth of the neural network: 13 convolutional layers and 16 convolutional layers, respectively. As the champion of the ImageNet Challenge in 2014, VGGNet still is one of the most popular networks.

ResNets [35] are designed with residual blocks, which solves the gradient vanish problem by appending a shortcut connection from input to output in each block. The use of residual block in ResNets makes training very deep neural networks possible. ResNet, with the advantage of much deeper networks, won the ImageNet Challenge in 2015.

Inception networks [37] make networks deeper and larger by parallel paths with 1 × 1, 3 × 3 and 5 × 5 convolutional filters, followed by max pooling layers. The features in different scales can be simultaneously extracted in the same layer. Hence, the feature extraction efficiency of inception networks is increased, which makes them faster than VGGNet.

DenseNet [36] creates a densely connected network in which the input of each layer is the stack combination of the outputs from all previous layers. DenseNet takes advantage of lower-level features and shared parameters so that the vanishing-gradient problem can be alleviated and network accuracy can be increased without performance loss.

(2) R-CNN series deep learning-based algorithms

The salient feature of R-CNN [43] methods is contained in two stages, namely region proposal and prediction. The objective of region proposal is to locate potential regions in the target image that may contain certain objects. In other words, it should have the highest possibility to predict specific targets in the generated regions by region proposal. In the following stage, a convolutional neural network is applied to make predictions in the above regions.

Mainstream region proposal methods include multi-scale combinatorial grouping (MCG) [44], constrained parametric min-cuts (CPMCs) [45] and region proposal networks (RPNs) [46]. MCG is a unified method that creates and combines fine-quality multi-scale regions. The initial step is to construct the image pyramid by supersampling and subsampling. For each layer in the image pyramid, single-scale segmentation is performed. The following operations of rescaling, alignment and combination are executed in order, and segmented regions are obtained. In general, segments are generated by solving CPMC with several parameters. A large number of features comprising 34 categories and related to three sets, i.e., graph, region and Gestalt properties, are extracted from segments. The above normalized features are ranked through random forests regression on the largest overlap with ground truth. Small sets of segments representing the objects in an image are acquired. The RPN, as a fully convolutional network, shares image convolutional features and instructs the following network in the region to detect, which can reduce region proposal cost and significantly improve detection accuracy. A unified detection network merged with RPN achieved state-of-the-art performance on public datasets, which makes RPN a competitive region proposal method.

(3) YOLO and SSD series deep learning-based algorithms

Single-stage methods, represented by YOLO [47,48,49] and SSD [50] series, remove the region proposal step and treat object detection as regression. Given the simplicity of one-stage methods, the number of trainable parameters and layers is significantly reduced. YOLO series with improvements, such as multiple scales, feature pyramids, fully connected layer abandon, relative location, binary cross entropy loss, etc., have achieved high accuracy without significant loss of detection speed. SSD series, the other representative one-stage method, applies the strategy of multi-layer detection, hard negative mining and data augmentation. Except for poor detection accuracy on small objects, SSD has achieved satisfying performance on various benchmark datasets.

(4) Deep learning algorithms for conveyor belt tear detection

Deep neural networks for general-purpose object detection are designed for common objects. With several special designs, these general-purpose object detection networks can be improved for conveyor belt defect detection. Because of the underground working environment in coal mines, the images captured by industrial cameras usually have the characteristics of high noise and low contrast. Hence, the feature extraction ability of backbone networks is essential for conveyor belt detection algorithms. As the depth of backbone networks increases, more semantic features can be acquired. The neural network cannot be too deep because of the existence of the gradient vanish problem in early days. To address this problem and make deep neural networks generic, a residual module has been widely used in recent research. YOLOv3 [49], as a general-purpose object detection algorithm, adopted Darknet53 as backbone network by five repeats of the residual block and contained the strategy of multi-scale feature extraction.

Some research focused on the above problems has been published, and several improved deep learning-based conveyor belt detection methods have been proposed. Liu et al. [51] designed an improved conveyor belt damage detection method based on YOLOv3 in which EfficientNet [52] was applied as backbone network instead of Darknet53, and a dataset containing damage images of conveyor belts was established. EfficientNet is characterized by compound scaling and best compromise between network depth, width and image resolution with limited resources. With compound scaling applied, limited computational resources can be maximumly used. Hence, the improved conveyor belt damage detection method in [51] shows excellent performance. A deep convolution network with special adaptability (adaptive deep convolutional network (ADCN)) was proposed by Qu et al. [53], and a dataset of diverse conveyer belt damage images was established. The backbone, neck and head of the deep neural network are specially designed according to the characteristics of conveyor belt damage images. With the special techniques of ResBlock, Mish activation, spatial pyramid pooling (SPP) [54] and feature pyramid networks (FPNs) [55], the improved ADCN method is more competitive than the compared SVM method. Targeting conveyor belt deviation failure, Yi et al. [56] adopted an inspection robot and a MobileNet-based deep neural network to extract region of interest (ROI), containing the belt edge and the exposed idler from original images. Hough line transform and template matching algorithms were applied to detect the belt edges. The combined hybrid method of deep learning and machine vision can efficiently detect ROI and belt deviation degree in real time. The core in ROI detector based on a deep neural network is called depthwise separable convolution (DSC), which consists of two pars, i.e., a depthwise convolution and a pointwise convolution. The DSC module separates normal convolution into a depthwise convolution and a pointwise convolution. Compared with normal convolution, DSC obtains similar receptive fields with a significantly smaller number of parameters—one third, approximately. Hence, based on these advantages, DSC is commonly applied in lightweight deep neural networks, e.g., Xception and MobileNet. A summary of deep learning methods presented in Table 4.

### 3.4. Next-Generation Detection Methods

With the development of high-performance sensors, new composite materials and cloud computing technology, the innovative methods of belt monitoring and damage detection have been designed and proven to be effective.

A conveyor belt monitoring system called Aura IQ, developed in Australia by Mining3/CSIRO, is based on optical fiber and cloud computing. The main principle of Aura IQ is monitoring the variation of laser pulses along a fiber optic cable which deployed along the length of a conveyor. High-performance computers monitor the laser pulses in real time. As long as the signal changes of laser pulses (caused by bearing or belt wear) are detected, Aura IQ analyzes these data, makes a quick decision and alerts workers. The deployment of the optical fiber-based conveyor belt monitoring systems can reduce the number of workers required and effectively avoid occupational injury.

### 3.5. Experimental Evaluation

**Figure 9:**
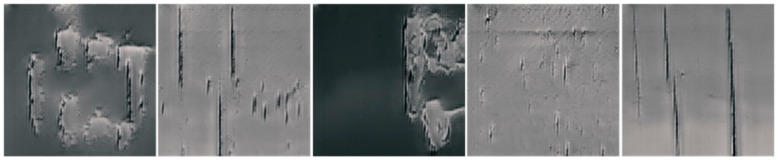
Sample conveyor belt damage images; damage types are cracks, tears and scratches.

In this section, various detection methods are compared with our custom belt damage dataset. Considering hardware limitations, some conveyor belt damage detection methods could not be tested. Hence, the compared algorithms and methods include support vector machine (typical classification algorithm); Faster R-CNN and YOLOv5 (typical deep learning algorithms); and Y. The belt damage dataset consists of three categories, i.e., cracks, tears and scratches, and each image may contain one or multiple damage points. The capacity of the dataset is 1092, and the number of crack, tear and scratch images is 212, 192 and 688, respectively. Some sample images are shown in Figure 9.

**Figure 10:**
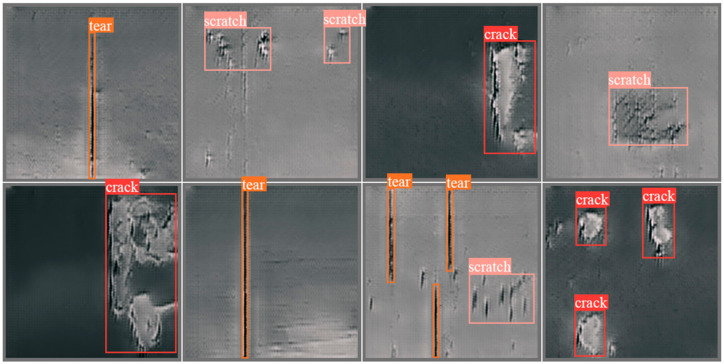
Visualization results contain anchor boxes and labels, showing that the obvious damage regions can be successfully detected. However, some small or inconspicuous damage regions are missed by detection algorithms.

YOLOv5 and Faster R-CNN represent for one-stage and two-stage deep learning-based object detection algorithms, respectively. YOLOX-s is designed for lightweight applications. The experimental results are described in Table 5 and Figure 10.

The experimental results show that all methods except for Faster R-CNN satisfy the real-time detection methods. Since these comparative algorithms are not specially optimized, the performance of all comparative algorithms can be enhanced. According to correlation theories, the factors influencing algorithm generalization are summarized as follows. (1) The selected algorithms focus on general purposes and were not optimized for belt damage detection. (2) For the traditional machine vision algorithms, some preprocessing procedure need to be done before the detections limits the speed of detection. (3) The custom dataset has fewer classes, and the acquisition condition of the custom dataset is different from that of the well-known public datasets. These reasons lead to better performance for most deep learning methods than identical methods trained and tested on public datasets. In general, the traditional machine vision algorithms are based on a combination of artificial feature extraction and certain classifiers. The essential problem is that the performance of certain algorithms depends on artificially designed features and engineering experience, which limits the development of machine vision algorithms. With the development of deep learning methods, a variety of network architectures have been proposed. The performance of deep learning algorithms is overwhelming and pleasurable. However, trained models are becoming bigger and more redundant, which is not suitable for limited computing resources, such as mobile applications. However, the inspiration from the experimental results is that deep learning-based detection algorithms have the potential to improve performance by improving feature extraction ability and modifying model architecture, loss function or training procedures.

## 4. Challenges and Solutions

### 4.1. Challenges for Image Preprocessing

In general, pixels in a normal image can be categorized into foreground, background and mixed pixels. Foreground pixels usually form the region where the desirable target is located, and background pixels make up areas that do not concern users. These two parts usually occupy 90% or more of the whole image. Mixed regions are significant for image segmentation. Since the “sharp boundary” or “soft boundary” determines the complexity of segmentation, image preprocessing procedures to obtain the desired “boundary” are essential for detection algorithms.

The working condition of conveyor belts for mines is underground for the most part. This means that there is no interference from sunlight at all, which is an advantage of setting up camera lights. However, working environment in mines is full of coal and ore dust, which could cause uneven brightness and has a negative impact on image acquisition by industrial cameras. However, with the progress of advanced technology, a variety of industrial cameras with hardware denoising ability and high-sensitivity chips can reduce the influence of dark, dusty and even moist working conditions to a certain extent. However, research has illustrated that the unprocessed images captured by industrial cameras still cannot satisfy the requirement of defect detection algorithms, and the procedure of image preprocessing is indispensable because of gray imbalance, noise, low contrast, etc. However, deep learning-based defect detection methods usually contain no steps of image preprocessing and feed image-label datasets directly into training networks. Image preprocessing procedures in most of machine vision-based literature we reviewed consist of image denoising, histogram equalization and contrast enhancement, which can increase the feature extraction ability and trained-model performance of algorithms.

Conveyor belt tear detection methods based on machine vision have developed from a singular reliance on visible light to a combination of visible light [27,28,29,31], hyperspectral [20,21,22,34], multispectral [23,24] and even audio-assisted [30] methods. No matter which methods are proposed, similar threshold algorithms are adopted in the image preprocessing step. The transfer conveyor belt tear features to several linear laser breakages and algorithms based on laser breakage location were proposed [32,33] to detect damaged belts, which greatly reduces the effect of acquired image quality on the performance of the proposed algorithm. However, image preprocessing steps, e.g., grayscale transformation and denoising, are still carried out linear laser images.

In summary, preprocessing is one of the most important steps in image processing algorithms. However, in contrast to images captured in other domains, images captured in dark and dusty underground environments cannot satisfy the requirement of defect detection because of low contrast and lots of noise. Thus, image preprocessing determines the final performance of detection algorithms to a large extent.

### 4.2. Challenges in Dataset Imbalance

In recent years, machine learning and deep learning methods based on pretrained models have gained attentions from researchers. The performance of supervised learning and deep neural networks is significantly dependent on the model used in the inference process, which is trained from a prepared dataset. He et al. [57] believe that the definition of data imbalance can be described as ratio of majority to minority distributed from 100:1 to 1000:1 in a large dataset. In the real world, problems caused by dataset imbalance, one of the most common dataset challenges, occur frequently.

As discussed in [58], data-level methods and algorithm-level methods are two general categories that tackle the data imbalance problem in traditional data or big data. For data-level methods, which can be classified as either oversampling or undersampling methods, are proposed to construct several training subdatasets. Data sampling is performed with a specially designed sampling approach or simply random selection. Random undersampling (RUS) [59], random oversampling (ROS) [60] and the synthetic minority oversampling technique (SMOTE) [61] are the most used data sampling approaches. For random oversampling methods, samples from the minority class are copied and extracted repeatedly in the oversampling process by some kind of algorithm [62]. Compared with ROS, random undersampling (RUS) methods construct various training subdatasets by applying random sample removal strategy. In contrast to ROS methods, SMOTE creates synthetic samples in feature domains instead of replacing in oversampling. Furthermore, SMOTE methods consider the k nearest neighbors in feature space and generate a new sample by interpolation strategy. Feature selection methods, which have been barely explored by scholars, are generally adopted to enhance classification performance [63] by extracting unique features for class discrimination.

Algorithm-level methods explore data classification at the algorithm level and can be classified into cost-sensitive methods and ensemble methods. Cost-sensitive methods apply more weight to misclassified instances in training process and select the most interesting samples. Ensemble methods consist of several parallel or serial classifiers, which output combined results from above classifiers. Ensemble methods are presented as bagging and boosting [64] types or hybrid methods, which have their own advantages. Bagging builds subclassifiers by training subsamples extracted from the whole dataset and combines the individual models into the final classification. Boosting implements the same procedure, which trains models from several individual subsamples extracted from the whole dataset, like bagging, and weights each classifier with adaptive value based on the misclassified ratio. Hybrid methods combine multiple data sampling methods with basic learning algorithms, e.g., naïve Bayes [65], and addresses currently known problems.

We believe that a certain class-balancing algorithm could not achieve best performance in all domain datasets because different characteristics are contained in different datasets. Fernandez et al. [66] conducted some experiments and examined data balancing in big data. The results show that RUS, ROS and SMOTE have significant performance differences in certain datasets. RUS and ROS have their own advantages in different configurations, whereas SMOTE performs the worst of the three.

Deep neural networks are trained with big data, which are usually much larger than traditional datasets, and acquire better performance because of their strong feature extraction capability. Although the methodologies for class equalization are similar between traditional datasets and big data, methods for addressing data imbalance in big data require specially designed algorithms. Data imbalance problems in the mining domain for deep learning methods can be classified into class imbalance and scale imbalance [67].

Class imbalance means a certain class in the dataset is over-represented. For the belt tear datasets, it refers to foreground- (tear part in belt image)-to-background (other objects in belt image) imbalance, which usually means background objects outnumber foreground objects. To address class imbalance problems, two typical sampling methods are frequently used: hard sampling and soft sampling methods. In general, the difference between hard sampling and soft sampling methods is the definition of bounding box (BB) contribution to the loss function. The term representing the BB contribution to the loss function is as follows:

(7) $$l_{BB}=\omega_{i}CE\left(p_{s}\right)$$

where $CE\left(*\right)$ is cross-entropy loss and $p_{s}$ represents confidence score of the ground truth class. For hard sampling methods, $\omega_{i}$ can be either 0 or 1. On the other hand, $\omega_{i}$ is a real number between 0 and 1 for soft sampling methods. The simplest hard sampling method is random sampling, which is applied in R-CNN detectors [43,46]. In the process of training RPN, 128 positive examples and 128 negative anchors are sampled randomly. Hard-sampling mining methods are based on the hypothesis that samples, which lead to high loss in loss function, contribute more to object detection performance. Single-shot detectors [50], as the first deep learning detector based on hard-sampling mining, take only the negative samples leading to the highest loss. In contrast to hard sampling methods, soft sampling methods assign the weight for each sample, and all samples in the dataset contribute to updating the model parameters. Focal loss [68], as the typical soft sampling parameter, dynamically weights examples as follows:

(8) $$\omega_{i}={\left(1-p_{s}\right)}^{\lambda }$$

where $p_{s}$ represents the confidence score of the ground truth class and λ is a constant. In [68], λ=2 makes a good compromise between performance and complexity. The gradient harmonizing mechanism (GHM) [69], another typical example of soft sampling, alleviates the variance of gradients derived from easy positive and negative examples. However, GHM assumes that easy examples lead to similar gradients, which has been proven to be useful for both classification and regression tasks.

On the other hand, scale imbalance occurs when positive instances appear in different scales and different quantities, which can be explained by the fact that tear parts are different in size and number for certain belt image datasets. Hence, the scale imbalance problem is inevitable because tear defects occur randomly as large regions or small spots. To address scale imbalance problems, we should review the basic detection procedure of deep neural networks. Early deep learning-based detectors [43] contained a backbone network and made predictions based on the last layer of the backbone network. However, the features extracted from the last layer of the backbone are restricted to a certain scale. In other words, the tear part whose size is beyond or within certain scale cannot be detected by the deep neural network. To address scale imbalance problems, a scale-diversity strategy, listed in Table 6, for input images or feature detection networks is applied.

Strategies of multi-scaled features, feature pyramid networks and image scaling pyramids in (Table 6) try to alleviate scale imbalance problems in different ways. Considering diverse features encoded in different scales, early research applied the multi-scaled features strategy and the designed deep neural networks extracted various scaled features from layers of backbone networks. To enhance feature extraction ability and mine semantic information buried among multiple layers of backbone networks, feature pyramid networks were proposed to merge low-level and high-level features. The concept of multi-scaled image pyramids is popular in machine vision-based methods because of its straightforward and simple theory. Since each layer of image pyramids fed into deep neural network cost computational time and memory, they would not be cost-efficient to employ in deep learning-based methods. Singh et al. [76] proposed scale normalization for image pyramids (SNIP) and discussed that multiple region proposal and detection networks can ensure no feature loss in training datasets.

Deep learning-based algorithms, a typical supervised learning method, possess great high-dimension feature extraction ability, which is based on prelabeled big datasets. However, limited to the mining industry, well-labeled datasets for conveyor belt defects do not exist, and deep learning-based algorithms cannot obtain stronger competition than traditional machine learning algorithms with artificially designed features. Different domain datasets, e.g., defect images of conveyor belts or features extracted by certain algorithms, have specific characteristics. The class of the conveyor belt image datasets can mainly be categories into normal belt, scratched surface, wear, cracked and torn belt, with unique corresponding visual features. Compared with cracks and tears, belt images of minor damage are predominant in conveyor belt datasets, and serious damage, e.g., cracks and tears, are rare but significant. Since the ratio of each class in conveyor belt datasets is uneven, classifiers trained by imbalanced datasets tend to predict the major categories and ignore the minor ones. To enhance the performance of deep learning-based algorithms, research and technologies such as transfer learning (TL) [77] and adversarial neural network (GAN) [78] target training with insufficient datasets and have been gaining more and more attention.

Transfer learning is another promising machine learning method that attempts to address data imbalance problems. TL methods aim at enhancing model performance based on transferring existing knowledge in the source domain to the target domain, which can reduce the dependency on the target domain dataset to some extent. Based on the above advantages, TL methods have been widely employed in medical image analysis [79,80], engineering [81], text processing [82], natural language processing [83] etc. Considering whether the existing dataset is labeled or not, TL can be classified into three categories, i.e., inductive, transductive and unsupervised transfer learning [77]. Based on the dataset distributions of source domain and target domain, TL approaches can be divided into homogeneous transfer learning and heterogeneous transfer learning. Considering the situation of the coal mining domain, dataset acquisition and labeling of conveyor belt defect images is extremely difficult and inconvenient. Hence, heterogeneous transfer learning from other similar domains to belt defect detection could be one possible solution to address data imbalance problems. Based on the hypothesis of Inception-v3 network possessing excellent feature extraction ability, Feng et al. [84] adopted a transfer learning strategy and improved the architecture of the Inception-v3 network in infrastructure damage detection. Gao et al. [81] tried to address the same problem of insufficient datasets. An improved VGGNet was proposed, and transfer learning was introduced. With several experiments, it was proven that the last two layers in a CNN network contain most high-level features for structural damage detection and classification. The surface defects of conveyor belts, i.e., scratches, cracks, tears, etc., are similar but different from those of injection-molded products, textiles or cold-rolled steel sheets. Hence, without modifying the architecture of deep neural networks and augmenting the dataset, transferring pretrained networks from other domains is one feasible approaches.

The concept of generative adversarial networks (GANs) was first proposed by Goodfellow et al. [78] in 2014 based on game theory. GANs consist of two networks, called generator and discriminator networks. The generator network tries to create the realest samples that can cheat the discriminator. At the same time, discriminator plays the role of judge to determine whether a given sample is artificial. Hence, a conflict exists between the generator and discriminator networks until the Nash equilibrium is achieved in the zero-sum game described by Pan et al. [85]. Architecture modification of deep neural networks based on GANs can be classified into three categories: (1) modified CNNs [86]; (2) conditional GANs [87,88]; and (3) autoencoders [89,90]. Modifying convolutional neural networks with the theory of GANs is a simple and straightforward way to implement GANs. Radford et al. [86] proposed a deep convolutional generative adversarial network (DCGAN), which introduces a deconvolution layer instead of the original fully connected layer in CNN. A conditional GAN was proposed in [87], and the conditional variable c was introduced in both the generator and discriminator, which makes the data generation process controllable. Autoencoders are another type of deep neural network, which has a process of input data encoding and reconstruction for output data. Makhzani et al. [89] merged the concept of GANs with autoencoding theory and proposed an adversarial autoencoder (AAE). Introducing GANs into defect detection for conveyor belt surfaces could alleviate data imbalance problems and enhance the performance of deep neural networks relating to GANs since GAN approaches extract low- and high-level features and generate new data based on existing datasets.

## 5. Conclusions

In this paper, we summarized the background and related knowledge of defect detection methods for conveyor belt surfaces in coal mining environments, including the basic structure of common conveyor belts, the several causes of conveyor belt tear, inspection system architecture and detection methods. The most common approaches consist of sensor-based, X-ray/spectrum-based and vision-based methods. Finally, the image preprocessing and data imbalance problems were investigated, and future research directions are discussed.

Industrial production in the field of coal mining requires automatic and intelligent inspection of production equipment. Hence, scholars and engineers have never stopped working on the research of automatic inspection and detection methods. In early days, the lack of high-performance processors and high-resolution cameras limited the development of non-destructive testing methods based on multiple sensors. These methods have the characteristics of simple installation, low cost and accuracy and high probability of false alarm. Therefore, defect defection for conveyor belt surfaces could not reach the goal of unmanned operation. Since the 21st century, without the limitation of hardware performance, more and more competitive methods have been proposed. To achieve the goal of automation and artificial intelligence in industrial production, vision-based detection methods are the most promising choice. In early 21st century, scholars proposed abundant machine vision-based defect detection methods with artificially designed features. In the last decade, because of high-level feature extraction ability, high performance and end-to-end network architecture, deep learning-based detection methods have been and will continue be the most promising approach. However, until existing shortcomings are overcome, deep learning-based methods cannot be widely applied. Future research should focus on the design and optimization of network architecture, efficient utilization of hardware resources, processing and preparation of datasets, etc.

## Figures and Tables

**Table 1:** Classification of conveyor belt defect detection methods.

Taxonomy	Devices	Theory Description and Advantages/Disadvantages
Sensor-based methods	Magnetic induction sensor, electromagnetic induction sensor	Convert belt damages into electromagnetic signals; then, analyze signal patterns to indirectly obtain the state of the conveyor belt. Simple principle, high cost and low precision.
X-ray/spectrum-based methods	X-ray emitter and receiver, industrial hyperspectral camera	X-ray penetrates conveyor belt and is captured by a special receiver, and the conveyor belt damage can be recognized by analyzing X-ray images. Hyperspectral cameras can image in infrared light, which decreases the influences of dusty and dark environments. X-ray-based methods can internally inspect the belt directly and precisely; complicated devices, high cost, harmful to humans. Spectrum-based methods are less influenced by environment; high cost, low precision.
Machine vision-based/deep learning-based methods	CCD, COMS or 3D industrial cameras	Industrial cameras take pictures of the conveyor belt surface in real time, which are simultaneously processed by a specially designed algorithm; not complicated devices, medium cost, complex algorithms.

**Table 2:** Comparison of X-ray/spectrum methods.

Method	Pros	Cons
X-ray [13,14,15,16,17,18,19]	Can detect internal damage of steel cord belt.	(1) Expensive and complicated equipment; (2) Requires large space to deploy; (3) X-ray is harmful to humans, and extra protection is required; (4) Instable detection.
Infrared [20] (one camera)	(1) Acquires infrared images; (2) Can detect early wear of conveyor belt.	(1) Based on image binarization and morphological, low robustness; (2) Uses special camera, poor portability.
Spectrum [21] (one camera)	(1) Acquires infrared images; (2) Obtains features in frequency domain.	(1) Domain transformation may lead to information loss; (2) Complicated computation.
Infrared [22] (two cameras)	(1) Novel optical path; obtains synchronous infrared and normal images; (2) Acquires extra information in fusion images.	(1) Direct image fusion; no information filtering.
Spectrum [23] (two cameras)	(1) Novel optical path; uses two infrared cameras to obtain different spectrum images; (2) Can detect belt tear in severe conditions.	(1) Uses expensive equipment; (2) Image resolution is low.
Spectrum [24] (two cameras)	Acquires images of different spectra; can obtain abundant useful features.	(1) Uses expensive equipment and requires large space to deploy cameras; (2) Complicated algorithm and computation.

**Table 3:** Comparison of machine vision methods.

Method	Pros	Common Cons
Segmentation [25,26,28,30]	(1) Based on image segmentation; (2) The logic of the algorithms is simple.	(1) Designs of artificial features and some of methods need to set special threshold, which leads to poor robustness; (2) Some algorithms contain complicated manually designed features; (3) Some methods adopt linear cameras to acquire high-resolution images; hence, the algorithm speed is limited and cannot realize real-time detection.
SSR [27]	Based on reflection image model and SSR algorithm to extract belt tear features.	
Classifier [29,31]	(1) These algorithms extract belt tear features and apply classic classifiers, which have stable performance; (2) Have made efforts to address poor robustness.	
Edge or corner features [32,33]	(1) Based on edge or corner features, which can focus on the region of belt tear; (2) Adopt linear cameras to obtain high-resolution images.	

**Table 4:** Comparison of machine vision methods.

Method	Pros	Cons
R-CNN	(1) Typical two-stage algorithm; after many improvements, algorithm is well developed and for applications that require high precision; (2)Based on region proposal networks; significantly improves detection precision.	(1) Region proposal networks make redundant bounding boxes, which leads to low speed; (2) The models are complex, and computational cost is high.
YOLO	(1) Simultaneously predicts object class and location as a regression process and gets rid of the region proposal stage, which simplifies the architecture and increases the speed; (2) Introduces multi-scale feature maps, which can enhance performance.	(1) The performance (except for speed) of YOLO series is worse than that of R-CNN series; (2) Anchors are fixed to a certain ratio; generalization is poor; (3) Poor performance for small object detection.
SSD	(1) A compromise between speed and precision; can achieve excellent performance in certain applications; (2) Multi-scale feature map fusion, which addresses poor robustness to a certain extent; (3) Less sensitive to the feature extraction ability of backbone networks than two-stage algorithms.	(1) Many hyperparameters need to be set properly; (2) Separated feature maps, which leads to complicated computation.

**Table 5:** Experimental results on custom belt damage dataset and public datasets in which the score threshold is set to 0.5, image resolution of the custom dataset is 416x416 and the GPU is NVIDIA RTX 2080s. For public datasets, models were trained on VOC2007 and VOC2012 training datasets tested on the VOC2007 testing dataset.

Method	Backbone	Custom Dataset		VOC2007 + VOC2012	
		FPS	mAP(%)@.5	FPS	mAP(%)@.5
Multi-SVM	Null	28.4	61.3	24.3	47.1
AdaBoost	Null	23.7	39.8	19.3	43.7
YOLOv5m	Focus+CSP	128	82.5	117	63.2
YOLOX-X	Modified CSPv5	57.4	78.4	55.8	65.3
SSD300	VGG16	59.1	81.7	57.4	72.6
Faster R-CNN	ResNet-101	7.4	86.4	6.2	74.9

**Table 6:** List of scale-diversity strategies and descriptions.

Strategy	Description
(a) No scale	Does not employ scale balancing strategy.
(b) Multi-scaled features [50,70,71]	Features extracted from different layers of backbone network are used to make predictions.
(c) Feature pyramid networks (FPN) [55,72,73,74]	Based on up-sampling or down-sampling methods; intermediate features extracted from adjacent layers of backbone network are merged to new features, which are used to make predictions.
(d) Scaled image pyramids [75,76]	Input image is scaled into different levels, and each scaled image is fed into the backbone network.

## Data Availability

Not applicable.
